# Seeding the Spine: Salmonella Java Discitis and Osteomyelitis After Traveller's Bacteraemia

**DOI:** 10.7759/cureus.108729

**Published:** 2026-05-12

**Authors:** Nazar Abdelgabar, Yahia Khalil, Layan Abu-Khalaf, Mohammad Khalil

**Affiliations:** 1 Gastroenterology and Hepatology, St Mary’s Hospital, Isle of Wight NHS Trust, Newport, GBR; 2 Internal Medicine, St Mary's Hospital, Isle of Wight NHS Trust, Newport, GBR; 3 School of Medicine, Royal College of Surgeons in Ireland, Dublin, IRL; 4 School of Medicine, Royal College of Surgeons in Ireland - Bahrain, Manama, BHR

**Keywords:** rare salmonella presentations, salmonella bacteremia, salmonella enterica discitis, travel-related infection, vertebral osteomyelitis

## Abstract

Non-typhoidal Salmonella infection usually causes self-limiting gastroenteritis, but a minority of cases are complicated by bacteraemia and extraintestinal focal infection. Vertebral discitis and osteomyelitis are rare complications, most often described in patients with diabetes, haemoglobinopathy, or immunosuppression. We describe a 67-year-old man with hypertension and previous percutaneous coronary intervention who attended the emergency department five times over six weeks with fever, rigors, diarrhoea, and worsening thoracic back pain after travel to Sri Lanka and the Maldives. Blood cultures grew Salmonella Java susceptible to ceftriaxone, ciprofloxacin, and co-trimoxazole. Initial CT of the chest, abdomen, and pelvis was unremarkable. CT angiography performed several weeks later showed mid-thoracic vertebral destruction and disc space change. MRI confirmed severe T6-T7 discitis and osteomyelitis with prevertebral soft tissue involvement of the adjacent oesophagus and azygous vein, together with periaortic enhancement from T5/T6 to T9. CT angiography found no aortitis. A repeat echocardiogram showed no evidence of infective endocarditis. Treatment required several antimicrobial adjustments, including prolonged intravenous ceftriaxone and later oral step-down to amoxicillin. Back pain improved substantially and serial radiographs showed no vertebral collapse. This case illustrates that Salmonella Java can seed the spine without classical immunocompromising risk factors and that the diagnosis is often delayed when persistent back pain is initially attributed to travel-associated gastroenteritis. Recurrent or unresolved symptoms after a confirmed Salmonella bacteraemia warrant early spinal MRI.

## Introduction

Salmonella species are Gram-negative bacilli responsible for both typhoidal and non-typhoidal infections. In immunocompetent adults, non-typhoidal disease is usually a self-limiting gastroenteritis that does not need hospital admission. A smaller number of cases become invasive, and once bacteraemia is present, the organism can seed bone, joint, or endovascular sites [1]. Travel to South Asia or sub-Saharan Africa is a recognised risk factor for acquiring non-typhoidal Salmonella, with returning travellers contributing a meaningful share of imported cases seen in high-income countries [2]. In adults with confirmed non-typhoidal Salmonella bacteraemia, the rate of extraintestinal focal infection has been reported as high as 39.5%, with mycotic aneurysm, pleuropulmonary infection, and spinal osteomyelitis the three most common foci [3]. Vertebral involvement is rare, reported in around 0.8% of all Salmonella infections (typhoidal and non-typhoidal serovars combined) [4], and is often diagnosed late because back pain is usually put down to musculoskeletal causes and the spine is not imaged until the patient comes back again [5,6].

Salmonella enterica serovar Paratyphi B var. L(+) tartrate+, commonly known as Salmonella Java, is a d-tartrate-fermenting biotype of Salmonella Paratyphi B that, unlike sensu stricto strains, usually causes a self-limiting gastroenteritis rather than enteric fever [7,8]. Reported reservoirs include tropical fish aquariums, reptiles, contaminated poultry, and dairy products, and infection has been linked both to sporadic exposure and to international travel [9]. S. Java is generally less invasive than other non-typhoidal serovars, but invasive disease and multidrug-resistant outbreaks have been described, and it accounts for a small but consistent fraction of imported salmonellosis in the United Kingdom and Europe [9,10].

Spinal salmonellosis is most often described in patients with haemoglobinopathy, diabetes, HIV infection, malignancy, or on immunosuppressive treatment, though it has been reported in patients with none of these [4-6,11]. The gastrointestinal phase frequently resolves before the vertebral focus becomes apparent, and that gap in the clinical picture is a consistent source of delay. The patient described here had no recognised immunocompromising condition. Following travel to South Asia, he developed Salmonella Java bacteraemia that was eventually confirmed to have caused thoracic discitis and osteomyelitis, with MRI also suggesting periaortic inflammatory involvement.

## Case presentation

The patient was a 67-year-old man with hypertension managed with ramipril and a myocardial infarction in 2022, treated with percutaneous coronary intervention. He had no diabetes, haemoglobinopathy, HIV infection, malignancy, or history of systemic immunosuppression, and was a non-smoker. His alcohol intake had been approximately 30 units of red wine per week. He stopped drinking in November 2025 after several weeks of feeling generally unwell, with no prior history of alcohol withdrawal symptoms or liver disease. He was taking omeprazole, though the documented indication was not available. He had carpal tunnel syndrome affecting both hands. The left hand had been treated surgically. The right hand had been managed with local corticosteroid injections at approximately two-yearly intervals, five injections in total over ten years, with the last dose given in 2024. He had never received oral corticosteroids. His typhoid vaccination was up to date, given his regular travel to South Asia.

From around 18 February to early March 2025, a small subcutaneous lump on his left foot broke down to form an ulcer, managed by the district nursing team without antibiotic therapy. From around April 2025, he had been aware of persistent lethargy.

He had travelled regularly to South Asia for many years, with frequent visits to both Sri Lanka and the Maldives. On his most recent trip, he returned to the United Kingdom on 24 October 2025. In Sri Lanka, he stayed in a hotel with a friend; in the Maldives, he stayed in a guesthouse and cooked for himself. His diet during travel included dried smoked tuna, chilli, coconuts, chapati, fish, and crab, but no oysters. He had no contact with reptiles or amphibians. His travelling companions ate similar food and had no gastrointestinal illness.

On 19 November 2025, he attended the emergency department with a two-day history of fever, rigors, metallic taste, nausea with retching, and dry cough. He had watery dark-brown diarrhoea four to five times daily with considerable flatulence. Thoracic back pain with spasms was also present, between the shoulder blades and extending towards the pelvis, beginning as a dull ache before becoming spasmodic. Examination found no focal cardiorespiratory or abdominal abnormality, but confirmed pyrexia. Laboratory values at this and the next attendance are given in Table 1. Chest radiograph showed heart size at the upper limits of normal with no consolidation or pleural effusion. A point-of-care COVID test and urine culture were both negative. Blood cultures taken on this occasion subsequently grew Gram-negative bacilli in both bottles. Intravenous co-amoxiclav was administered in the emergency department. The hyponatraemia was attributed in part to high alcohol intake, and he was discharged with oral co-amoxiclav, the course of which was later extended to 13 days in total.

**Table 1:** Laboratory values at the first two emergency department attendances. Abnormal values are in bold. Reference ranges are those used in UK adult clinical biochemistry. Values not documented at the given attendance are shown as Not recorded.

Parameter	Reference range	19 November 2025	22 November 2025
C-reactive protein	<5 mg/L	~200	253
White cell count	4.0–11.0 × 10⁹/L	10	10.6
Sodium	133–146 mmol/L	130	130
Potassium	3.5–5.3 mmol/L	Not recorded	3.3
Bilirubin	<21 µmol/L	28	24
eGFR	>90 mL/min/1.73 m²	Not recorded	85

He returned on 22 November 2025 with ongoing watery diarrhoea, excessive flatulence, severe back muscle spasms, fever, and rigors. Blood cultures from the first attendance had grown Salmonella species, susceptible to co-trimoxazole, azithromycin, ceftriaxone, and intravenous amoxicillin, with intermediate susceptibility to oral amoxicillin. He received intravenous fluids with potassium supplementation, intravenous paracetamol, and liquid morphine for back pain. Point-of-care testing for COVID and influenza A and B on 24 November was negative. He was discharged with advice on oral hydration and continuation of antibiotics.

On 28 November 2025, he returned with a poor appetite and reduced oral intake. Azithromycin 500 mg once daily was started, given the confirmed Salmonella diagnosis. He was admitted to the acute assessment unit the same day, as his condition had not improved. During that admission, which lasted five days, he received intravenous ceftriaxone alongside oral azithromycin 500 mg once daily. He developed a mild rash over the forehead and postauricular areas during this period. Azithromycin was stopped, and the rash resolved within three days. HIV testing on 29 November was negative; a respiratory panel including RSV, influenza A and B, and SARS-CoV-2 on 30 November was also negative. He improved and was discharged on 3 December 2025. Following discussion with the consultant microbiologist, azithromycin was restarted at discharge; the rash had been mild, had fully resolved, and azithromycin remained the preferred oral agent given the susceptibility profile and available alternatives at that stage.

On 11 December 2025, the patient attended the phlebotomy unit as an outpatient. Staff noted he appeared unwell; he was taken to same-day emergency care and subsequently to the emergency department, where he received paracetamol for back pain. He was admitted to the ward, and a PICC line was inserted for antibiotic administration. He was alert with a GCS of 15, heart sounds were normal, lung fields were clear, and his abdomen was soft and non-tender. Blood cultures grew Salmonella Java. Susceptibility testing confirmed sensitivity to ciprofloxacin, gentamicin, co-trimoxazole, ceftriaxone, and intravenous amoxicillin, with intermediate susceptibility to oral amoxicillin. Blood culture results and the antimicrobial susceptibility profile from both positive sets are given in Table 2. HIV testing and a respiratory viral panel were both negative. An ECG showed no QTc prolongation.

**Table 2:** Positive blood culture sets and antimicrobial sensitivity profile. The 19 November set was reported initially as Salmonella species and the serovar was confirmed later; the 11 December set was reported as Salmonella Java on isolation. Only agents tested on each occasion are shown.

Parameter	First set — 19 November 2025	Second set — 11 December 2025
Organism	Salmonella species (later identified)	Salmonella Java
Ceftriaxone	Sensitive	Sensitive
Ciprofloxacin	Not reported on this set	Sensitive
Gentamicin	Not reported on this set	Sensitive
Co-trimoxazole	Sensitive	Sensitive
Azithromycin	Sensitive	Not reported on this set
Amoxicillin (intravenous)	Sensitive	Sensitive
Amoxicillin (oral)	Resistant	Resistant

**Figure 1:**
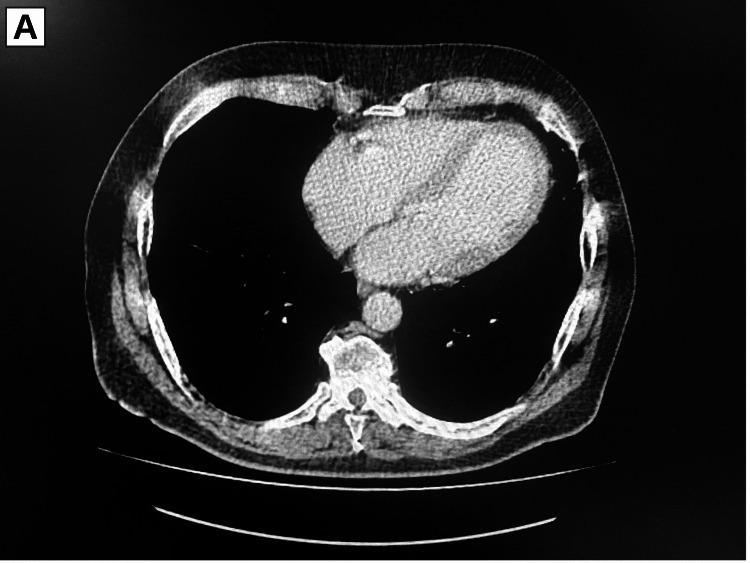
Initial CT Imaging Initial CT of the chest, abdomen, and pelvis. Axial thoracic section on lung windows. The study found no acute intra-abdominal pathology and no mycotic aneurysm; incidental findings were bilateral lower lobe atelectasis, a sub-centimetre left upper lobe pulmonary nodule in keeping with post-infectious change, small bilateral renal cysts, and prostatic calcifications.

Following discussion with the consultant microbiologist, intravenous ceftriaxone was restarted, and ciprofloxacin was introduced alongside it. Imaging was arranged to identify a deep infective focus and exclude endovascular complications. CT of the chest, abdomen, and pelvis showed no acute intra-abdominal pathology and no mycotic aneurysm. Incidental findings included bilateral lower lobe atelectasis, a sub-centimetre left upper lobe pulmonary nodule consistent with a post-infectious or inflammatory process, small bilateral renal cysts, and prostatic calcifications; none suggested an alternative infective focus (Figure 1). Upper abdominal ultrasound showed no hepatobiliary pathology, with a small right pleural fluid collection noted incidentally.

Ciprofloxacin was subsequently stopped after he developed a further rash. Ceftriaxone was continued as monotherapy at an escalated dose of 4g once daily for one week.

**Figure 2:**
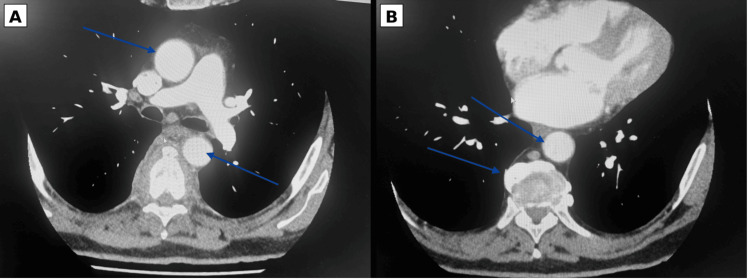
CT Angio CT angiography of the aorta, 23 December 2025. (A) Axial section at the aortic arch and great vessels. The aorta is well opacified with no wall thickening or periaortic fat stranding. (B) Axial section at the level of the affected vertebral segment, with the opacified descending thoracic aorta adjacent to the vertebral body. There is interval disc height loss and pre- and paravertebral soft tissue change in keeping with discitis and osteomyelitis (reported at T7–T8), but no mycotic aneurysm, aortitis, or epidural abscess. The affected vertebral level was reported as T7–T8 on CT angiography and T6–T7 on MRI; this difference reflects the lower spatial resolution of CT for early endplate change rather than progression of disease

CT angiography of the aorta on 23 December 2025 demonstrated interval vertebral destruction with disc height loss and pre- and paravertebral soft tissue change at the T7-T8 level, consistent with discitis and osteomyelitis. No aortic wall thickening, periaortic fat stranding, epidural abscess, or aortitis was identified (Figure 2).

**Figure 3:**
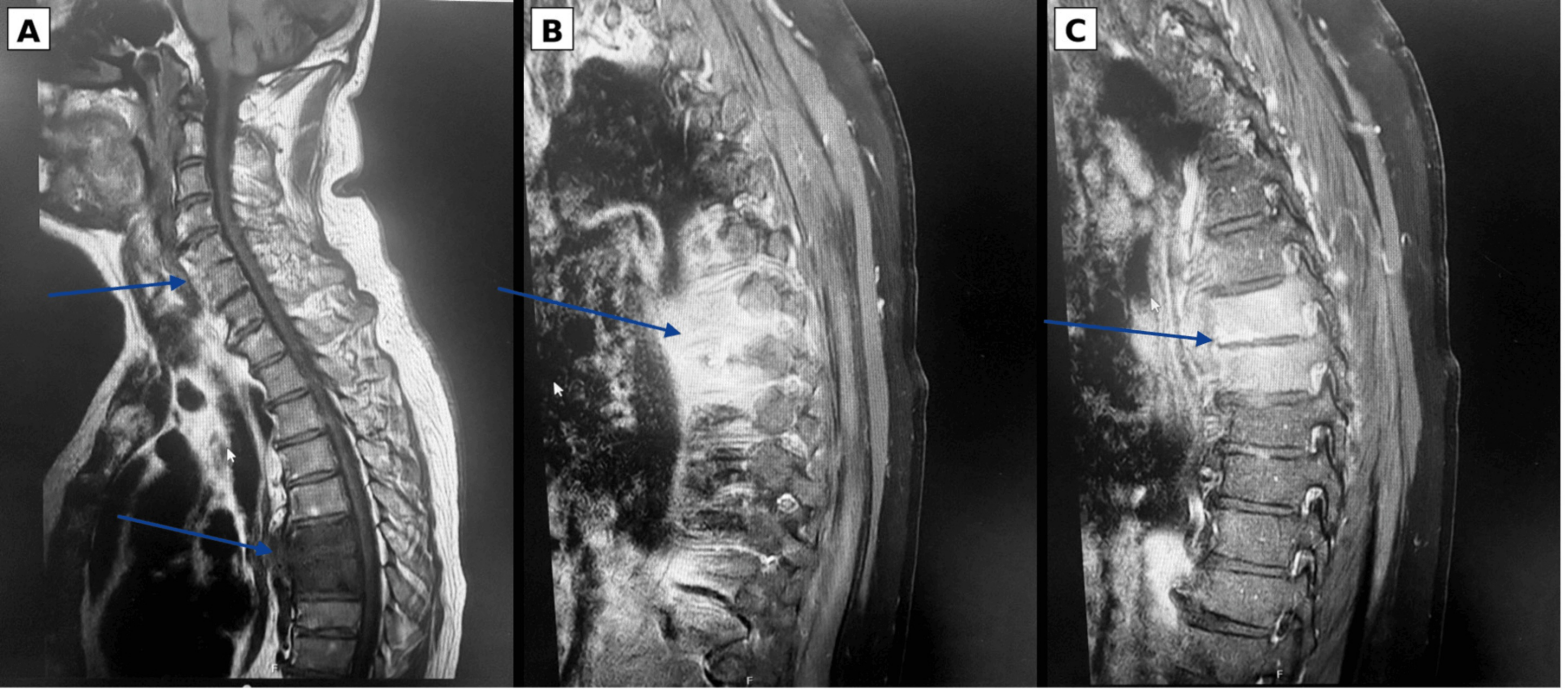
MRI Whole-spine MRI with contrast, 24 December 2025. Sagittal views. (A) Overview through the cervicothoracic spine, with loss of disc height and altered marrow signal at T6–T7. (B) Fluid-sensitive sequence through the thoracic spine, showing marked marrow oedema in T6 and T7, destruction of the intervening disc, and prevertebral inflammatory signal. (C) Post-contrast sequence with fat saturation, with avid enhancement of both vertebral bodies and the intervening disc, plus enhancement of the adjacent prevertebral soft tissues extending anteriorly. Appearances were those of severe T6–T7 discitis and osteomyelitis. The affected vertebral level was reported as T7–T8 on CT angiography and T6–T7 on MRI; this difference reflects the lower spatial resolution of CT for early endplate change rather than progression of disease

**Figure 4:**
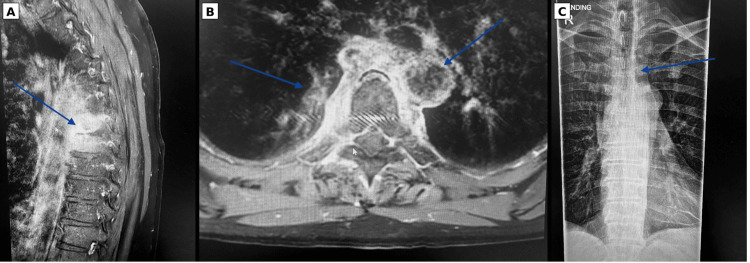
MRI & X-ray Additional MRI findings and follow-up imaging. (A) Post-contrast sagittal thoracic MRI with fat saturation. A strip of enhancement is seen anterior to the vertebral column from approximately T5/T6 to T9, in keeping with periaortic inflammatory change. This was not apparent on the CT angiography performed the previous day. (B) Axial post-contrast image at the level of the affected segment, with prevertebral soft tissue involvement abutting the oesophagus and azygous vein. There was no epidural extension or cord compression. (C) Thoracic spine anteroposterior radiography, 28 January 2026. Persistent T6–T7 disc space narrowing with endplate erosion, with reduced vertebral body heights at T6, T7, and T8. Spinal alignment was preserved and there was no vertebral collapse.

MRI of the whole spine with contrast on 24 December 2025 confirmed severe T6-T7 discitis and extensive osteomyelitis of both vertebral bodies, with marked marrow oedema and post-contrast enhancement (Figure 3). Inflammatory change extended into the adjacent prevertebral soft tissues, involving the oesophagus and azygous vein. Enhancement was also noted around the descending thoracic aorta from approximately T5/T6 to T9, though this had not been evident on CT angiography (Figure 4). There was no prevertebral or epidural collection, cord compression, or nerve root compromise. Multilevel degenerative changes were present throughout the spine without significant neural compromise. The report recommended urgent spinal and vascular review.

Echocardiography on 19 December 2025 showed preserved left ventricular systolic function with an ejection fraction of 60-65%, a dilated left atrium, trivial to mild mitral regurgitation, mild tricuspid regurgitation with an estimated pulmonary artery systolic pressure of 29 mmHg and could not fully exclude endocarditis. A repeat study on 30 December 2025 confirmed an ejection fraction of 55%, slight posterior mitral valve prolapses, no significant mitral regurgitation, and no evidence of infective endocarditis. The thoracic aorta was not well visualised on either study.

Both the spinal and vascular surgical teams were involved in management, and a vascular multidisciplinary meeting was convened. Specialist tropical medicine advice was sought; an initial recommendation of 12 weeks of antibiotic treatment was subsequently revised to a total duration of six months, given the extent of bone and soft tissue involvement. The spinal team advised serial thoracic spine radiographs at 12 and 26 weeks to monitor for vertebral collapse. Atorvastatin was withheld for deranged liver function tests and tamsulosin was withheld for low blood pressure. Vitamin D deficiency was identified, and supplementation was started. He had lost approximately 10 kg since mid-November 2025, with associated muscle mass loss; he was advised on a high-protein diet and graded exercise.

The thoracic spine radiograph on 30 December 2025 showed mid-thoracic disc space narrowing and endplate erosion at T6-T7, reduced disc height, background osteoporosis, and degenerative changes with osteophytes throughout the mid and lower thoracic spine. Healing was in progress but not yet complete.

He was discharged on 9 January 2026 to continue intravenous ceftriaxone 2g once daily via the outpatient parenteral antimicrobial therapy service. On 28 January 2026, he attended as an outpatient and received a single dose of ceftriaxone 4g. The thoracic spine radiograph on the same day showed persistent T6-T7 disc space narrowing, erosion of the inferior endplate of T6 and superior endplate of T7, and reduction in vertebral body heights at T6, T7, and T8, with maintained spinal alignment and no vertebral collapse (Figure 4C).

Following further microbiology discussion, ceftriaxone was transitioned to oral co-trimoxazole 960 mg three times daily. Blood tests showed mild renal impairment attributed to the antibiotic, and the frequency was reduced to twice daily. Co-trimoxazole was stopped after ten days. Following further specialist microbiology and tropical medicine advice, oral amoxicillin 1g three times daily was commenced on 5 March 2026, with the dose selected to optimise tissue concentrations despite the intermediate susceptibility to oral amoxicillin on prior testing and planned to continue until June 2026.

Back pain diminished considerably during admission and continued to improve after discharge. Appetite recovered, muscle mass began to return with adherence to dietary and exercise advice, and no neurological deficit was documented at any point.

## Discussion

The spinal diagnosis was not established until the fifth attendance, nearly four weeks after back pain was first documented. Blood cultures had grown Salmonella on two separate occasions before imaging was directed at the spine. The back pain was present from the outset but was not investigated independently at early attendances; each presentation was dominated by what appeared to be a resolving enteric illness, and the back pain was not investigated as a separate problem until bacteraemia was confirmed on a second set of cultures.

Vertebral osteomyelitis due to Salmonella is uncommon. Published cases show that back pain and fever tend to dominate once spinal seeding has occurred, while gastrointestinal symptoms are frequently absent or already resolved by the time the vertebral diagnosis is confirmed [4-6,11,12]. The condition is most often described in patients with haemoglobinopathy, diabetes, or immunosuppression, though it has been documented in patients without any of these [4-6,11,12].

Several factors that may have predisposed this patient to invasive disease were present, although causality cannot be inferred from a single case. Each is supported in the literature as a risk factor for invasive non-typhoidal Salmonella infection, but the contribution of any one factor in this presentation cannot be quantified. Older age, atherosclerotic vascular disease, travel to an endemic region, chronic alcohol excess, and a history of local corticosteroid injections to the right hand were all present. Chronic alcohol use impairs both innate and adaptive immunity, including neutrophil function, T-lymphocyte responses, and gut-associated lymphoid tissue, and is recognised as a contributor to invasive bacterial infection [13]. Long-term omeprazole use was also noted. Proton pump inhibitor therapy increases the risk of enteric infection including non-typhoidal salmonellosis, with the proposed mechanism being a reduction in the gastric acid barrier [14,15]; the individual contribution of omeprazole here cannot be determined. HIV was negative and there was no diabetes, haemoglobinopathy, or oral corticosteroid use.

CT angiography excluded aortitis and mycotic aneurysm. MRI performed the following day characterised the T6-T7 focus in considerably more detail, showing marrow oedema, disc destruction, and prevertebral inflammatory involvement extending to the oesophagus and azygous vein, none of which had been apparent on CT. The periaortic enhancement from T5/T6 to T9 seen on MRI was not present on CT angiography, which found no aortic wall thickening or fat stranding. No vascular intervention was required. CT angiography is generally more sensitive than MRI for structural aortic wall pathology such as mycotic aneurysm, intramural haematoma, and dissection, while contrast-enhanced MRI is more sensitive for early or subtle aortic wall inflammation and periaortic oedema [16]. The discrepancy between the two studies in this region most likely reflects this difference in modality sensitivity rather than progression of disease over the 24 hours between scans.

Antimicrobial management required several adjustments throughout the course. Azithromycin was stopped early for a mild drug rash, then restarted at discharge on microbiologist advice once the rash had fully resolved, given its favourable susceptibility profile and the limited oral alternatives available at that point. Ciprofloxacin, introduced during the December admission alongside ceftriaxone, was stopped when a further rash developed, and ceftriaxone was escalated as monotherapy. Co-trimoxazole subsequently required dose reduction for renal impairment and was replaced by high-dose oral amoxicillin for the outpatient phase, selected on specialist advice to optimise tissue penetration despite intermediate susceptibility on prior testing. Specialist tropical medicine input extended the planned treatment duration from 12 weeks to six months, reflecting the extent of bone and soft tissue involvement. Such adjustments are not uncommon when prolonged pathogen-directed therapy is required in older adults with multiple comorbidities, and close microbiological and biochemical review was needed throughout. Current guidance for native vertebral osteomyelitis recommends frequent review during prolonged antimicrobial courses [17].

The choice of high-dose oral amoxicillin (1 g three times daily) for the consolidation phase, despite resistant susceptibility on prior testing, was made on specialist tropical medicine and microbiology advice. By that point, most alternatives had been exhausted: azithromycin and ciprofloxacin had each caused rash, and co-trimoxazole had caused renal impairment. The dose was selected to optimise systemic exposure during prolonged consolidation therapy, and the regimen was tolerated well enough to continue the planned six-month course.

Repeated re-presentation despite antibiotic treatment should prompt a review of the diagnosis rather than continuation of the same management plan. In this case, back pain that persisted through multiple attendances eventually led to spinal imaging and a diagnosis that had not been apparent from the enteric presentation alone.

## Conclusions

Salmonella Java bacteraemia caused vertebral discitis and osteomyelitis in a patient without the immunocompromising conditions classically associated with invasive Salmonella disease. Persistent back pain, recurrent fever, or ongoing systemic deterioration after enteric Salmonella infection should prompt blood cultures and early contrast-enhanced MRI of the spine, which remains the imaging modality of choice for native vertebral osteomyelitis. Where imaging raises concern for adjacent endovascular or structural involvement, multidisciplinary input and prolonged pathogen-directed therapy guided by susceptibility testing and specialist advice are needed, and treatment duration may need to be extended according to imaging and clinical response.
